# Urban Mangroves Under Threat: Metagenomic Analysis Reveals a Surge in Human and Plant Pathogenic Fungi

**DOI:** 10.3390/pathogens14080759

**Published:** 2025-08-01

**Authors:** Juliana Britto Martins de Oliveira, Mariana Barbieri, Dario Corrêa-Junior, Matheus Schmitt, Luana Lessa R. Santos, Ana C. Bahia, Cláudio Ernesto Taveira Parente, Susana Frases

**Affiliations:** 1 Laboratório de Biofísica de Fungos, Instituto de Biofísica Carlos Chagas Filho, Universidade Federal do Rio de Janeiro, Rio de Janeiro 21941-902, Brazil; julianamartins@biof.ufrj.br (J.B.M.d.O.); marianabrito@biof.ufrj.br (M.B.); dariojunior@biof.ufrj.br (D.C.-J.); matheusschmittmr45@gmail.com (M.S.); 2 Laboratório de Bioquímica de Insetos e Parasitos, Instituto de Biofísica Carlos Chagas Filho, Universidade Federal do Rio de Janeiro, Rio de Janeiro 21941-902, Brazil; luanalrs.bio@gmail.com (L.L.R.S.); anabahia@biof.ufrj.br (A.C.B.); 3 Laboratório de Estudos Ambientais Olaf Malm, Instituto de Biofísica Carlos Chagas Filho, Universidade Federal do Rio de Janeiro, Av. Carlos Chagas Filho s/n, Bloco G0, Sala 60, Subsolo, Rio de Janeiro 21941-902, Brazil; cparente@biof.ufrj.br; 4 Rede Micologia RJ, Fundação Carlos Chagas Filho de Amparo à Pesquisa do Estado do Rio de Janeiro, Rio de Janeiro 21941-902, Brazil

**Keywords:** fungal pathogens, urban mangroves, metagenomic analysis, Guanabara Bay

## Abstract

Coastal ecosystems are increasingly threatened by climate change and anthropogenic pressures, which can disrupt microbial communities and favor the emergence of pathogenic organisms. In this study, we applied metagenomic analysis to characterize fungal communities in sediment samples from an urban mangrove subjected to environmental stress. The results revealed a fungal community with reduced richness—28% lower than expected for similar ecosystems—likely linked to physicochemical changes such as heavy metal accumulation, acidic pH, and eutrophication, all typical of urbanized coastal areas. Notably, we detected an increase in potentially pathogenic genera, including *Candida*, *Aspergillus*, and *Pseudoascochyta*, alongside a decrease in key saprotrophic genera such as *Fusarium* and *Thelebolus*, indicating a shift in ecological function. The fungal assemblage was dominated by the phyla *Ascomycota* and *Basidiomycota*, and despite adverse conditions, symbiotic mycorrhizal fungi remained present, suggesting partial resilience. A considerable fraction of unclassified fungal taxa also points to underexplored microbial diversity with potential ecological or health significance. Importantly, this study does not aim to compare pristine and contaminated environments, but rather to provide a sanitary alert by identifying the presence and potential proliferation of pathogenic fungi in a degraded mangrove system. These findings highlight the sensitivity of mangrove fungal communities to environmental disturbance and reinforce the value of metagenomic approaches for monitoring ecosystem health. Incorporating fungal metagenomic surveillance into environmental management strategies is essential to better understand biodiversity loss, ecological resilience, and potential public health risks in degraded coastal environments.

## 1. Introduction

Mangroves are vital coastal ecosystems found predominantly in tropical and subtropical regions, performing key ecological functions such as nutrient cycling, carbon sequestration, coastal protection, and serving as nurseries for diverse aquatic and terrestrial species [1,2,3]. These environments maintain a delicate ecological balance through interactions among diverse microbial communities, including fungi that act as saprotrophs, pathogens, and symbionts [4,5]. While pristine mangroves typically host functionally diverse fungal assemblages adapted to stable conditions of pH, salinity, tidal flux, and oxygen gradients [2], anthropogenic disturbances combined with climate change threaten to disrupt this balance, favoring pathogenic fungi and reducing functional diversity [2].

Urbanization and industrial activities impose multiple stressors on mangroves, including chemical contamination from heavy metals, organic pollutants, and nutrient overload, altering physicochemical parameters such as pH, salinity, dissolved oxygen, and sediment composition [6,7]. These changes intensify eutrophication and reduce habitat quality, fostering conditions conducive to opportunistic fungi with pathogenic potential, such as *Candida*, *Aspergillus*, and *Fusarium* [6,8,9]. Recent research on mangrove sediments from Techeng Isle, China, has uncovered strong associations between heavy metal concentrations and fungal communities. For example, chromium (Cr), lead (Pb), and vanadium (V) were positively linked to the genus *Eutypella*, while showing negative correlations with genera such as *Cladosporium* [10]. In urban estuaries, zinc (Zn) and chromium (Cr) have emerged as major factors influencing shifts in microbial communities, with sulfate-reducing bacteria displaying negative correlations with metal pollution [11]. Studies across several mangrove sites in southern China have identified zinc and lead as the main contaminants and have found that bacterial genera like *Fusibacter* and *Syntrophorhabdus* are significantly associated with heavy metal levels [12]. Collectively, these findings—supported by broader reviews—demonstrate that persistent heavy metal contamination can profoundly shape the composition and diversity of microbial communities in ecosystems such as mangroves and estuaries [13]. These complex interactions between heavy metals and microbes have important implications for ecosystem health and the development of bioremediation strategies. In contrast, clean mangroves exhibit balanced fungal communities where symbiotic and saprophytic species maintain nutrient cycling and ecological stability [2,4].

Studies in impacted mangroves, including Rio de Janeiro’s Guanabara Bay, highlight the prevalence of pollutants such as mercury, lead, chromium, and hydrocarbons, which alter microbial community structure and favor resistant pathogenic fungi [7,14]. This highly urbanized region encompasses 16 municipalities of the Rio de Janeiro Metropolitan Area, housing approximately 16 million inhabitants, with about 6 million residing in Rio de Janeiro city alone [15]. The area receives over 2.8 million international tourists annually, many of whom visit coastal areas around the bay, including Niterói and Rio’s coastline, intensifying human exposure risks [16]. The extensive anthropogenic pressure has dramatically transformed the landscape: of the original 260 km^2^ of mangroves, only about 82 km^2^ remain, with coastal areas now dominated by urban and industrial development rather than natural ecosystems [17]. The degradation of water quality through contamination and altered salinity regimes, driven also by climate change-induced sea level rise and altered tidal patterns, exacerbates stress on microbial assemblages, selecting for fungi with enhanced virulence and antifungal resistance [18,19,20]. Experimental evidence demonstrates that such environmental stressors modulate fungal gene expression related to pathogenicity and resistance mechanisms, including the overexpression of regulators like Upc2p, which confers resistance to azole antifungals in *Candida albicans* [21,22].

Pathogenic fungi in mangroves represent a dual threat: ecologically, by disrupting functional microbial diversity and nutrient cycling, and from a public health perspective, as reservoirs of opportunistic infections, especially in immunocompromised populations [6,9,23]. Recent studies highlight the concerning relationship between environmental contamination and pathogenic fungi. Heavy metal pollution in ecosystems can affect fungal growth and induce stress responses, yet many pathogenic fungi and oomycetes show high tolerance to heavy metals [24]. Urban mangrove sediments have been found to harbor diverse yeast species, including potentially pathogenic *Candida* strains resistant to multiple antifungal drugs [9]. Anthropogenic impacts on mangroves have been linked to increased abundance of heavy metal resistance genes in microbial communities [25]. Furthermore, pathogenic *Candida* species have been isolated from plastic pollutants in aquatic environments, displaying drug resistance, thermotolerance, and virulence [26]. These findings suggest that contaminated environments may serve as reservoirs for drug-resistant fungal pathogens, posing potential risks to human health and highlighting the need for increased surveillance and interdisciplinary research from a One Health perspective. Moreover, the presence of thermotolerant fungi such as *Aspergillus fumigatus* and *Candida tropicalis* in eutrophic, polluted environments illustrates the synergistic effect of warming temperatures and pollution on pathogenic fungal proliferation [8,27].

While saprotrophic fungi remain crucial for organic matter decomposition and nutrient recycling in mangrove soils, the increasing dominance of pathogenic and resistant fungal taxa reflects a functional shift with potential consequences for ecosystem resilience and health [4,28]. Symbiotic fungi, such as mycorrhizal species, may persist in impacted environments, but their roles can be compromised under chemical and physical stress [29]. These microbial shifts underline the importance of environmental variables—pH fluctuations, salinity changes due to altered tidal regimes, and oxygen depletion—as critical selective forces modulating fungal community structure and function [30].

Despite their ecological and health significance, comprehensive metagenomic studies of fungal communities in urban mangroves, especially focusing on pathogenic fungi under combined anthropogenic and climatic stress, remain scarce in Brazil and globally [31,32]. This gap limits the understanding of ecological risks and public health implications, as well as the development of effective conservation and management strategies [31,32,33].

This study aims to fill this gap by using a metagenomic approach to characterize fungal communities in urban mangrove sediments under environmental stress, focusing on the identification of pathogenic fungi and their relationship with altered physicochemical parameters (pH, salinity, oxygen levels) and climate-related changes. By comparing impacted mangroves with less disturbed sites, the research tests the hypothesis that anthropogenic contamination combined with climatic alterations selects for pathogenic and resistant fungal taxa, leading to functional reconfiguration of microbial communities with implications for ecosystem health and human risk.

## 2. Materials and Methods

### 2.1. Site Selection

Soil and sediment samples were collected in August 2024 from the margins of Guanabara Bay, Rio de Janeiro, Brazil, specifically from a mangrove area in the northeastern region of the bay that receives direct inputs of sewage waste and solid debris. The central sampling point was georeferenced at 22.79523° S and 43.20333° W. This site exemplifies urban coastal degradation, as the natural mangrove ecosystem is continuously exposed to untreated domestic effluents and accumulated garbage from surrounding urban settlements. The northeastern region of Guanabara Bay is part of the Metropolitan Region of Rio de Janeiro, which encompasses 16 municipalities and has an estimated population of approximately 16 million people, with about 6 million residing in the city of Rio de Janeiro alone [34]. While there are no specific data for the annual number of tourists visiting the bay itself, the city of Rio de Janeiro received over 2.8 million international tourists in 2024, many of whom visit coastal areas of the bay, such as Niterói and the Rio coastline. Historically, most of the bay’s coastline was covered by mangroves and natural areas. Currently, of the approximately 260 km^2^ of original mangrove coverage, only about 82 km^2^ remain, and agricultural activity along this coastal strip is virtually nonexistent due to intense urbanization and industrialization [17]. Guanabara Bay itself is a highly urbanized coastal zone with a well-documented history of significant environmental impacts, suffering from severe pollution due to untreated sewage, industrial discharges, and maritime activities [35]. This contamination poses serious risks to marine biodiversity and human health, with research revealing the presence of toxic metals [36] antifouling paint residues [37], and electronic waste pollutants in the ecosystem [38]. Soil samples were collected from an exposed intertidal area, away from the direct influence of the rhizosphere. Field observations—such as water saturation, muddy texture, sulfide odor, dark coloration, and high organic content—led to the classification of the soil as a Gleysol, following the World Reference Base for Soil Resources (WRB/FAO) guidelines) [39]. This soil type is characteristic of tropical mangrove environments, marked by anoxic conditions and regular tidal influence.

Due to the high degree of urbanization and industrialization in the Guanabara Bay region, all remaining mangrove areas are subject to some level of anthropogenic impact. As a result, it was not possible to include a truly undisturbed (pristine) mangrove site as a control for direct comparison. Furthermore, comparing our results with mangroves from distant, less-impacted regions would introduce confounding variables related to geography, climate, and local environmental conditions. Therefore, this study was designed to assess the fungal community structure and the presence of potential pathogenic taxa in a highly urbanized mangrove, serving as a sanitary alert for environments under intense anthropogenic pressure, rather than to compare pristine and impacted sites.

The sampling area is representative of the typical mangrove vegetation found in the northeastern region of Guanabara Bay, which is generally dominated by *Avicennia schaueriana* and *Laguncularia racemosa*. Although a detailed botanical survey was not conducted as part of this study, field observations confirmed the presence of these species in the vicinity of the sampling sites. All samples were collected from exposed intertidal sediments, away from the direct influence of the rhizosphere, in order to minimize the effect of root-associated microbial communities and focus on the broader sediment environment.

### 2.2. Sample Collection

Three sampling sites were selected along the Corredor Esportivo, located in Ilha do Governador, Rio de Janeiro, Brazil. The first sample was collected at the mouth of a sewage discharge Site 1: −22°47′41″ S, 43°12′05″ W, while the second site, Site 2: −22°47′35″ S, 43°12′10″ W, was located approximately 100 m away, in an area positioned along the mangrove extension toward the marine coast of Guanabara Bay. At each site, three replicate composite samples were collected. Each composite sample was formed by homogenizing three independent subsamples (triplicates) collected under mid-tide conditions using sterile, autoclaved spatulas. Approximately 50 g of surface sediment (0–3 cm depth) was collected per subsample and then combined to form the composite and placed into labeled, transparent, sterile containers (5–6 cm diameter, 8–12 cm height) with red caps. Samples were transported in sealed cardboard boxes and delivered to the laboratory within one-hour post-collection.

### 2.3. Sample Storage and Processing

Upon arrival, samples were stored at −80 °C to preserve microbial DNA integrity. Before DNA extraction, samples were thawed at room temperature under sterile conditions to prevent DNA degradation and contamination. The three replicates from each site were homogenized into a composite sample representative of each collection point.

### 2.4. DNA Extraction

Genomic DNA was extracted following a modified phenol-chloroform protocol. A 5-mL volume of material was pulverized using liquid nitrogen and homogenized with 500 μL of extraction buffer (50 mM Tris-HCl, 20 mM EDTA, 2% SDS, and 150 mM NaCl) and 4 μL of RNase A (10 mg/mL). The homogenate was added to a 1.5-mL tube and incubated at 65 °C for 30 min with intermittent mixing by inversion every 8 min to ensure complete cell lysis. After incubation, samples were centrifuged at 14,000 rpm for 10 min and the supernatant discarded. 350 μL of 3 M ammonium acetate was added to the pellet. The mixture was incubated on ice for 30 min to precipitate proteins and polysaccharides, followed by centrifugation at 14,000 rpm for 10 min. To the resulting supernatant, 700 μL of phenol:chloroform (1:1) was added, gently inverted, and incubated on ice for 5 min with periodic shaking. After centrifugation at 14,000 rpm for 10 min, the aqueous phase was collected and mixed with 500 μL of cold isopropanol until DNA precipitation was visible. The precipitated DNA was incubated on ice for 10 min, pelleted by centrifugation at 14,000 rpm for 10 min, and washed with 1 mL of cold 70% ethanol. After centrifugation and removal of ethanol, the DNA pellet was rehydrated in 50 μL of nuclease-free water. DNA quality and concentration were assessed by 1.5% agarose gel electrophoresis using undiluted and diluted samples at a ratio of 1:10.

### 2.5. Amplification and Sequencing

Fungal rRNA ITS regions were amplified using two primer sets: ITS1F/ITS2 for the ITS1 region and ITS3/ITS4 for the ITS2 region. PCR conditions included initial denaturation at 94 °C for 3 min; 30 cycles of 94 °C for 30 s, 57 °C for 30 s, and 72 °C for 30 s; with a final extension at 72 °C for 5 min. The libraries were prepared with sequencing adapters and purified. Sequencing was performed on the Illumina NovaSeq 6000 platform using paired-end reads. From two samples, a total of 159,761 raw paired-end reads were generated; after quality filtering, 141,263 clean reads remained, with a minimum of 70,585 and an average of 70,632 reads per sample.

### 2.6. Sequencing Platform and Read Type

The Illumina NovaSeq 6000 platform (Illumina, San Diego, CA, USA) generated paired end (PE) reads targeting the ITS1 and ITS2 rDNA regions. This approach improves sequence accuracy and taxonomic inference. Between 79,000 and 159,000 raw reads were obtained per region per sample [40].

### 2.7. Raw Data Processing

Initial quality filtering was performed with Trimmomatic v0.33 (sliding window: 50 bp; min Q-score: 20). Primer sequences were removed using Cutadapt v1.9.1 (max mismatch: 20%, min coverage: 80%). USEARCH v10 was used to merge PE reads (min overlap: 10 bp; min similarity: 90%; max mismatch: 5 bp). Chimeric sequences were removed using UCHIME v8.1. Quality metrics, including read count, sequence length, and chimera ratio, were tracked, ensuring > 99% sequencing coverage. Diversity metrics such as rarefaction curves, Shannon index, and rank-abundance plots confirmed sequencing depth adequacy.

### 2.8. TGS Data Processing and Quality Control

Raw subreads were processed to generate Circular Consensus Sequencing (CCS) reads using SMRT Link v8.0 (≥5 passes; accuracy ≥ 90%). Primer detection and demultiplexing were done with lima v1.7.0. CCS reads were filtered for expected ITS region lengths (300–1000 bp), and primers were removed using Cutadapt v2.7 (error rate: 20%). Chimeras were identified with UCHIME. Throughout the TGS workflow, accuracy, length distribution, and non-chimeric proportions were continuously assessed.

### 2.9. Bioinformatics Analysis

Raw sequences were processed using Trimmomatic v0.33 and Cutadapt v1.9.1, followed by the DADA2 pipeline in R for denoising, chimera removal, and ASV generation with single-nucleotide resolution. Taxonomic annotation employed the UNITE database through Bayesian classification and BLAST (v9.0) alignment. Only high-confidence assignments were retained, and classification to the species level was performed when possible. A relative abundance matrix of ASVs was created for each sample to infer community structure.

### 2.10. Microbial Diversity Analysis

Fungal composition was characterized from the kingdom to the species level. Alpha diversity was assessed using Chao1, ACE, Shannon, and Simpson indices. Beta diversity was analyzed via QIIME2 and R to compare community structures.

### 2.11. Statistical Analyses

Fungal ecological roles were predicted using the FUNGuild tool, which categorized taxa into saprotrophs, symbiotrophs, and pathotrophs, facilitating interpretation of ecological interactions and potential health risks associated with each site.

## 3. Results

### 3.1. Diversity and Sequencing Metrics

#### 3.1.1. Rarefaction and Community Evenness

**Figure 1:**
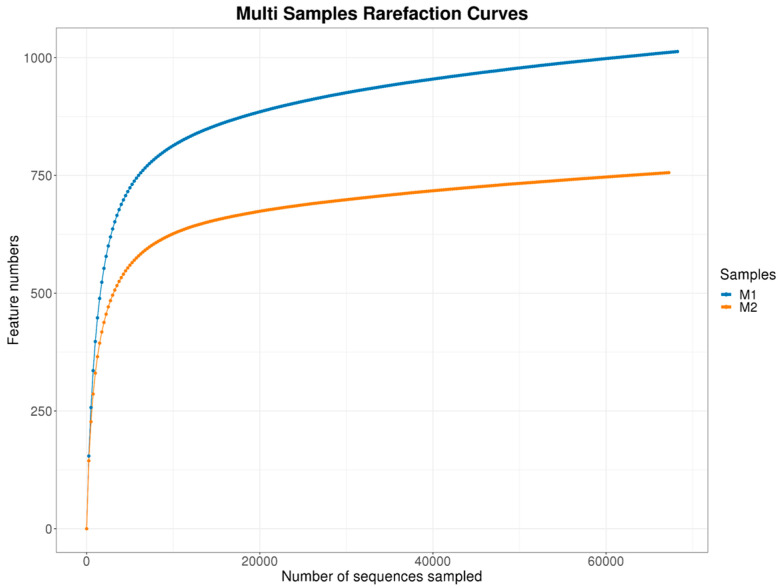
Rarefaction curves for fungal communities from sediment samples collected in an impacted mangrove. Both samples show a trend toward plateauing, indicating that the sequencing depth was likely sufficient to capture most of the fungal diversity present. The curve illustrates the relationship between the number of sequences sampled and the observed number of ASVs, reflecting the microbial richness within the studied environment.

The rarefaction curve shown in the Figure 1 demonstrates a plateau trend in both samples, indicating that the sequencing depth was sufficient to capture most of the fungal diversity present in the environment. The relationship between the number of sequences analyzed and the observed number of operational taxonomic units ASVs reflects the local microbial richness. The vertical bars associated with the curve represent confidence intervals, suggesting stability in diversity detection as the number of reads increases. These results reinforce the reliability of the metagenomic analysis applied to the studied mangrove sediment.

**Figure 2:**
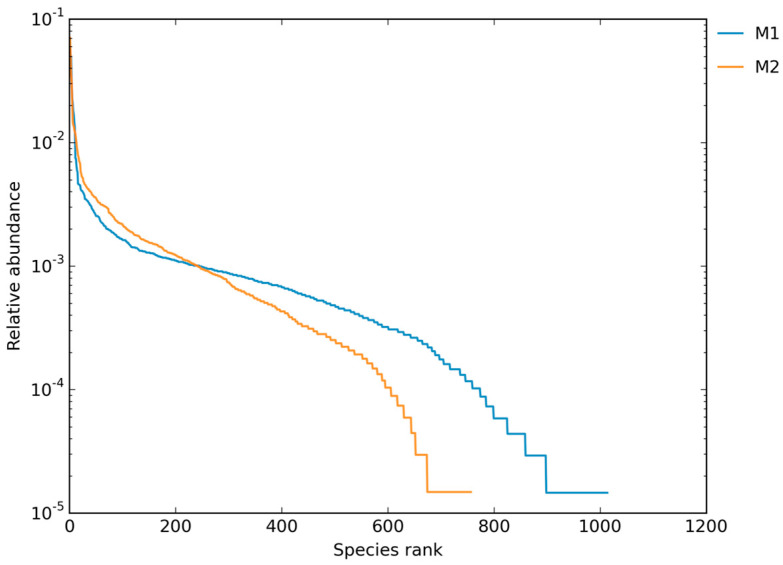
Rank-abundance curve of pathogenic fungi in mangrove sediments affected by anthropogenic and climatic impacts. The gentle slope of the curve indicates a relatively even distribution of fungal taxa, reflecting a diverse and well-distributed fungal community despite ongoing adverse environmental pressures. These results highlight the resilience and importance of pathogenic fungi in the sediment ecosystem dynamics under human influence and climate change.

The rank-abundance curve showed (Figure 2) a gentle gradient, suggesting a relatively even distribution of fungal taxa in the sediment samples. These findings indicate a rich and well-distributed fungal community, although influenced by adverse environmental conditions arising from continuous anthropogenic pressures.

#### 3.1.2. ASV/OTU Richness and Read Counts

The metagenomic analysis of fungal communities revealed a total of 1769 ASVs and 135,626 sequences (reads) from sediment samples collected in a single impacted mangrove. The sample with the highest genetic richness presented 1013 ASVs and 68,281 sequences, while the other sample resulted in 756 ASVs and 67,345 sequences, reflecting the fungal diversity and spatial heterogeneity that can occur even within a single ecosystem subject to anthropogenic pressures (Table 1).

#### 3.1.3. Alpha Diversity

**Figure 3:**
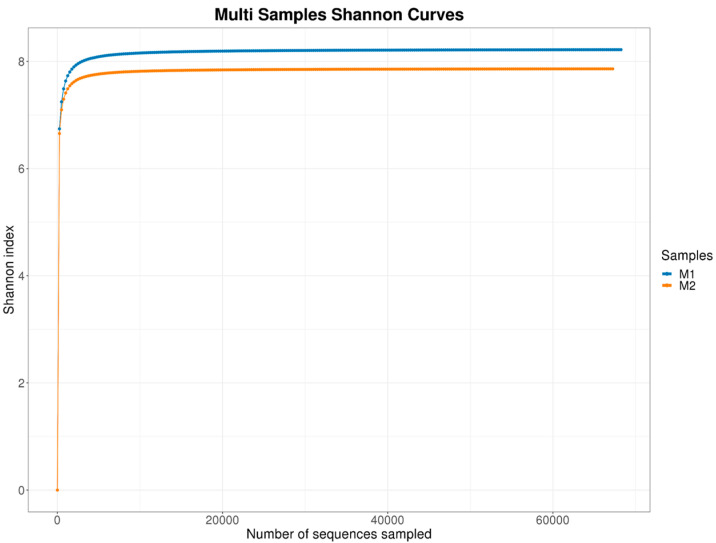
Sample M1 showed slightly higher diversity compared to M2, reflecting possible local microbial heterogeneity within the same impacted urban mangrove environment.

Alpha diversity was assessed using the Shannon index (Figure 3), which showed high and comparable values between samples, indicating high fungal diversity. Shannon values ranged from 7.46 to 8.21. Good’s coverage values (>99%) confirmed adequate sequencing depth to capture most fungal species.

### 3.2. Taxonomic and Functional Characterization

#### 3.2.1. Phylum-Level Distribution

**Figure 4:**
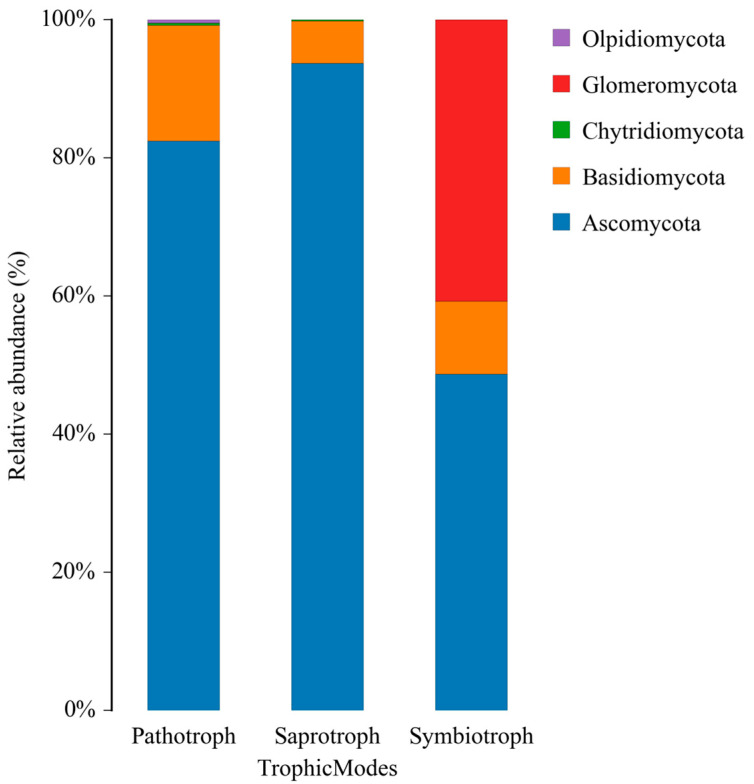
Distribution of major fungal phyla by trophic mode in impacted mangrove sediments. *Ascomycota* dominated pathotrophic and saprotrophic fungi, while *Glomeromycota* stood out among symbionts. *Basidiomycota* showed lower contributions across all groups.

As illustrated in Figure 4, members of the phylum *Ascomycota* exhibit overwhelming dominance among both pathotrophic (82.47%) and saprotrophic (93.71%) fungal communities. This marked prevalence underscores the phylum’s remarkable ecological plasticity, reflecting its dual capacity to function as a key agent in both pathogenesis and the decomposition of organic substrates. *Basidiomycota* was the second most abundant phylum in these groups, albeit with a significantly lower contribution (16.73% in pathotrophs and 6.11% in saprotrophs). In contrast, symbiotrophic fungal communities exhibited a more diverse phylum distribution, with *Ascomycota* still being the most prevalent (48.73%), but with a notable and substantial contribution from *Glomeromycota* (40.77%)**,** reflecting its crucial role in mycorrhizal associations. *Basidiomycota* was also present among symbiotrophs (10.50%). Phyla such as *Chytridiomycota* and *Olpidiomycota* showed very low abundances or absence, indicating their limited or no role in these specific trophic modes within the present dataset.

#### 3.2.2. Class-Level Functional Profiles

**Figure 5:**
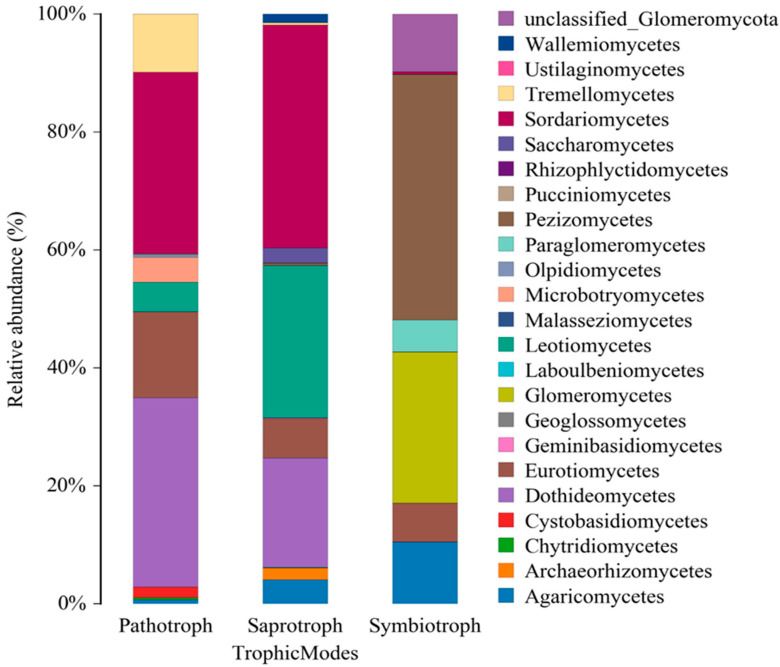
Relative abundance of fungal classes by trophic mode in impacted mangrove sediments. Pathotrophs were dominated by *Dothideomycetes* and *Sordariomycetes*, while saprotrophs were mainly represented by *Sordariomycetes* and *Leotiomycetes*. These patterns reflect microbial adaptations to environmental and anthropogenic stresses.

**Figure 6:**
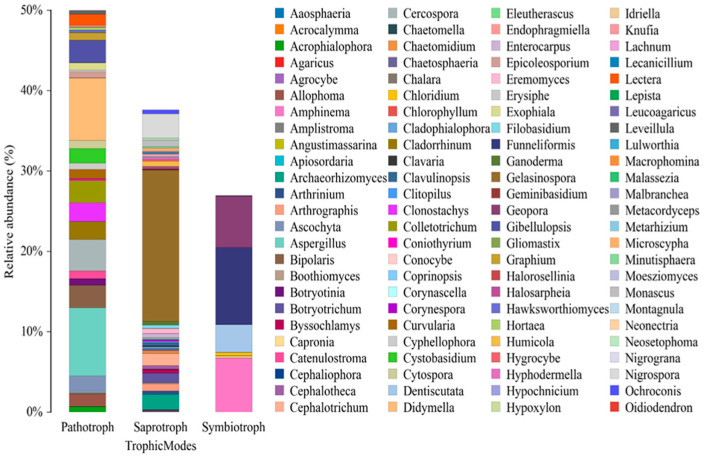
Pathotrophic, saprotrophic, and symbiotrophic fungal genera were identified in mangrove sediments affected by anthropogenic impacts. Among these, *Aspergillus* and *Didymella* stood out as the most abundant pathotrophic types, suggesting potential risks to native hosts. Additionally, other pathogenic genera may also play a role in the adaptation of fungal communities to stressed environments.

Metagenomic analysis of fungal communities in mangrove sediments reveals distinct taxonomic and functional profiles influenced by environmental pressures (Figure 6). Pathotrophic fungi, mainly *Dothideomycetes* (32.10%), *Sordariomycetes* (30.82%), *Eurotiomycetes* (14.58%), and *Tremellomycetes* (9.81%), are highly prevalent in stressed environments, potentially threatening ecosystem health. Saprotrophic fungi, essential for nutrient cycling, are dominated by *Sordariomycetes* (37.78%) and *Leotiomycetes* (25.79%), while symbiotrophic fungi, such as *Pezizomycetes* (41.69%) and *Glomeromycetes* (25.59%), though less abundant, remain important for symbiotic relationships. Shifts in these fungal groups due to pollution, climate change, or other disturbances may compromise sediment stability and ecosystem resilience (Figure 5).

The results revealed that *Dothideomycetes* (32.10%) and *Sordariomycetes* (30.82%) were the most abundant pathotrophic fungi in mangrove sediments, followed by *Eurotiomycetes* (14.58%) and *Tremellomycetes* (9.81%). The substantial presence of potential fungal pathogens is particularly concerning in mangrove ecosystems already under pressure from pollution, deforestation, and climate change. Such an imbalance in the fungal community may pose significant threats to mangrove vegetation, aquatic fauna, and overall ecosystem stability. Although saprotrophic and symbiotrophic fungi were also identified, the dominance of pathotrophs underscores the importance of monitoring disease dynamics and assessing the resilience of these vulnerable environments (Figure 5).

### 3.3. Pathogenic Potential and Ecological Implications

#### Fungal Genera and Trophic Modes

The detailed results of the fungal genus presented in the Figure 6, showing the composition in mangrove sediments with an emphasis on trophic modes, reveal a complex interplay among pathogens, saprophytes, and symbionts shaped by anthropogenic impacts. Among pathotrophic fungi, various genera were identified, with notable presences of *Aspergillus* (8.48%), *Didymella* (7.78%), and *Plectosphaerella* (5.06%), suggesting a broad range of potential threats to native hosts. Genera such as *Clonostachys* (2.33%), *Colletotrichum* (2.76%), *Bipolaris* (2.83%), and *Cercospora* (3.90%) also contribute to the pathogenic profile, indicating the capacity of these fungi to exploit niches in a stressed environment (Figure 6). The occurrence and abundance of these pathogens in mangrove sediments are particularly concerning, as they can be exacerbated by factors like pollution, deforestation, and altered hydrological regimes induced by climate change. Such conditions may increase the susceptibility of mangrove organisms to infections, compromising the health of local flora and fauna and the resilience of these vital ecosystems. While saprotrophic genera like *Gelasinospora* (18.81%), *Thelebolus* (22.24%), and *Preussia* (8.48%), and symbionts like *Tuber* (35.27%) and *Funneliformis* (9.58%) are also prevalent, the clear dominance of pathogenic genera within their respective trophic category demands further in-depth investigation into their potential ecological impact and mitigation strategies in vulnerable mangroves.

### 3.4. Functional Ecology of Fungal Communities

#### Guild Composition

Functional analysis based on the FUNGuild database revealed a predominance of fungi with saprotrophic trophic mode (41.62%), followed by pathotroph-saprotrophs (34.59%) and mixed trophic combinations (23.8%). The significant presence of fungi with pathogenic potential, combined with the abundance of unidentified groups, indicates an ongoing ecological restructuring of the community influenced by sediment contamination and associated environmental pressures. The occurrence of mixed trophic combinations—fungi capable of adopting more than one nutritional strategy—suggests a high degree of functional plasticity within the community. This plasticity may represent an adaptive response to environmental stressors, allowing fungi to exploit diverse resources and persist under changing or adverse conditions. Such functional diversity can contribute to the resilience of the fungal community but may also reflect shifts in ecosystem processes and interactions resulting from anthropogenic impacts.

## 4. Discussion

Recent studies have demonstrated that anthropogenic disturbances, including heavy metal pollution, eutrophication, oil spills, and sewage discharge, significantly impact fungal communities in mangrove ecosystems [10,41]. These environmental pressures are known to alter microbial diversity and community structure, with key physicochemical factors such as pH, total phosphorus, and heavy metals playing crucial roles in shaping both bacterial and fungal assemblages. In some disturbed mangrove sites, unexpected changes in fungal diversity have been observed, challenging previous assumptions about the relationship between contamination and community dynamics [42]. In some disturbed mangrove sites, unexpected changes in fungal diversity have been observed, challenging previous assumptions about the relationship between contamination and community dynamics. Physicochemical factors, such as pH, total phosphorus, and heavy metals, play crucial roles in shaping bacterial and fungal communities [10,41]. Oil spills and sewage pollution were identified as drivers of fungal community composition in highly polluted mangrove sites [43]. Interestingly, fungal endophytes from disturbed sites demonstrated remarkable phosphate solubilization capabilities, suggesting potential adaptations to elevated nutrient levels [42]. These findings highlight the complex relationships between environmental disturbances and fungal communities in mangrove ecosystems, emphasizing the need for further research to understand and preserve these vital habitats.

Although we did not directly measure organic carbon, nitrogen, pH, or heavy metal concentrations in our samples, our interpretations are supported by extensive published data and environmental assessments of Guanabara Bay, which consistently report high levels of pollution, nutrient enrichment, and altered physicochemical parameters in the region [7,35,36]. These well-documented environmental stressors are known to shape microbial community structure and function in urban mangroves. We recognize that future studies integrating direct physicochemical measurements with metagenomic analyses will be essential to further elucidate the complex interactions between environmental factors and microbial diversity in these ecosystems.

While *Ascomycota* and *Basidiomycota* were the dominant phyla in our samples, this pattern is commonly observed in soils and sediments from a wide range of climatic and natural zones. Our analysis, however, also revealed ecologically relevant shifts in community structure, including an increase in potentially pathogenic genera (such as *Aspergillus*, *Candida*, *Pseudoascochyta*, *Didymella*, *Plectosphaerella*, *Colletotrichum*, *Bipolaris*, and *Cercospora*) and a reduction in important saprotrophic groups (such as *Fusarium*, *Thelebolus*, and *Gelasinospora*), reflecting the impact of urban pollution on fungal community function. Beyond the dominant phyla, we detected several non-dominant and rare fungal taxa, including *Glomeromycota* (notably among symbiotrophs), and a considerable fraction of unclassified fungal ASVs. Although these groups represented a small fraction of the total community, their presence suggests a diverse and functionally complex fungal assemblage, even in highly impacted urban mangrove sediments. Rare taxa may play important ecological roles, such as participating in specialized nutrient cycles, contributing to resilience under environmental stress, or acting as reservoirs of genetic and metabolic diversity [44,45].

Regarding the low abundance of *Chytridiomycota* and *Olpidiomycota*, this finding is consistent with the highly anthropogenically impacted nature of the studied mangrove. The area receives considerable pollutant loads (such as urban sewage, heavy metals, and nutrient input), creating stressful environmental conditions that likely suppress sensitive fungal lineages such as chytrids and olpidiomycetes, which typically predominate in more stable and less contaminated aquatic environments [46]. Additionally, methodological factors such as primer specificity and DNA extraction efficiency may have contributed to the underrepresentation of these groups in our dataset. Similar patterns have been reported in other studies of polluted or urbanized mangrove systems. These considerations highlight the need for further research using complementary approaches to fully capture the diversity and ecological roles of rare fungal taxa in mangrove sediments [47].

Recent studies have further highlighted the complex interactions between soil fungi, heavy metals, and environmental factors. For example, zinc concentration in soil has been shown to significantly affect fungal colony-forming units, with *Aspergillus* and *Penicillium* species dominating contaminated soils [48]. Heavy metals can impact fungal growth, morphology, and sporulation, but many fungi exhibit high tolerance through various detoxification mechanisms [24]. In arid farm soils of Peru, fungal communities were found to be influenced by both soil properties and heavy metals, with an unusually high proportion of pathotrophs [49]. Moreover, climate change and environmental pressures may be selecting for fungi with increased thermotolerance and virulence factors, potentially leading to the emergence of new human pathogens. Species of *Cryptococcus*, *Aspergillus*, *Penicillium*, *Candida*, and *Scedosporium* have been identified as emerging threats in this context [50]. Consistent with these findings, our study identified genera such as *Aspergillus*, *Candida*, *Fusarium*, *Curvularia*, *Cladosporium*, and *Pseudoascochyta* as potential human pathogens, while *Colletotrichum*, *Didymella*, *Plectosphaerella*, *Bipolaris*, and *Cercospora* were recognized as potential plant pathogens. These results underscore the importance of monitoring fungal communities in polluted and climate-impacted environments, given their significant ecological and public health implications.

The ability of certain fungal genera to adapt to adverse conditions is well documented. For example, genera such as *Aspergillus*, *Fusarium*, *Curvularia*, and *Candida* are known for their tolerance to acidic and contaminated environments, as well as for possessing antifungal resistance genes such as CDR and MDR transporters [21,51,52]. The literature also reports that fungal endophytes from disturbed mangrove sites demonstrate remarkable phosphate solubilization capabilities, suggesting potential adaptations to elevated nutrient levels and eutrophic conditions [43]. This ecological plasticity allows these fungi to persist and even thrive under continuous environmental pressures, often resulting in a functional reconfiguration of the community [51,53,54,55].

The functional reorganization of fungal communities in response to anthropogenic stressors has important ecological implications. In preserved mangroves, saprotrophic fungi such as *Gelasinospora*, *Thelebolus*, and *Preussia*, as well as symbionts like *Tuber* and *Funneliformis*, play essential roles in nutrient cycling, soil formation, and plant health, contributing to the resilience and natural recovery of these environments [43]. However, in contaminated mangroves, the dominance of pathogenic and opportunistic fungi may compromise these ecosystem services, leading to reduced functional diversity and increased vulnerability to further disturbances [43,54,55]. The literature emphasizes that the loss or reduction of beneficial functional groups can undermine the ecological balance and long-term sustainability of mangrove ecosystems [43].

Beyond ecological consequences, the expansion of pathogenic and resistant fungal groups in anthropized mangroves raises significant public health concerns. Genera such as *Aspergillus*, *Candida*, *Fusarium*, *Curvularia*, and *Cladosporium* are frequently associated with clinical infections, particularly in immunocompromised individuals [56,57,58,59,60,61]. The ability of these fungi to persist in polluted environments, form biofilms, and resist antifungal agents complicates both environmental management and clinical treatment [57,60]. The literature underscores the importance of understanding the ecology and pathogenic mechanisms of these fungi to develop effective prevention and control strategies, especially as environmental changes and human activities continue to influence their prevalence and virulence [46,61].

The patterns observed in Brazilian mangroves are consistent with findings from other urbanized coastal environments. Studies conducted in impacted areas such as Guanabara Bay [62] and international research [53,63,64] report similar trends, including the dominance of *Ascomycota* in polluted environments, community segregation by trophic modes, and increased presence of pathogenic fungi in areas subjected to anthropogenic pressure. These similarities reinforce the hypothesis that urban mangroves are becoming hotspots not only of microbial diversity but also of potential risks to human health and ecological balance [62,64].

Given the complex interplay between environmental stressors and fungal community dynamics, the literature highlights the urgent need for ongoing research, continuous microbiological monitoring, and the implementation of effective conservation policies [62,64]. Understanding how anthropogenic pressures shape microbial diversity and function is essential for developing strategies to preserve the ecological integrity and public health value of mangrove ecosystems. The integration of molecular, ecological, and epidemiological approaches will be crucial for advancing knowledge and informing management actions in these vital coastal habitats.

We acknowledge that, although samples were collected away from the direct influence of the rhizosphere, the composition of mangrove vegetation may still exert indirect effects on sediment microbial communities through the release of phytoncides or other bioactive compounds. Therefore, we cannot completely exclude the possibility that plant-microbe interactions, including potential antagonism, may have contributed to the observed patterns of microbial diversity. Future studies including a detailed botanical survey and targeted rhizosphere sampling would help to further clarify these interactions.

## 5. Conclusions

The metagenomic analysis of fungal communities in urban mangrove sediments revealed a taxonomically rich yet functionally altered microbiota strongly influenced by environmental contamination and anthropogenic pressures. The dominance of phyla such as *Ascomycota* and *Basidiomycota*, alongside fungal taxa with known pathogenic potential and resistance traits, underscores the adaptive response of these communities to stressors like urban sewage, industrial effluents, heavy metals, and pharmaceutical residues.

The significant prevalence of genera including *Candida*, *Aspergillus*, and *Fusarium*, associated primarily with pathotrophic and sapro-pathotrophic lifestyles, indicates the emergence of ecologically resilient fungal assemblages that pose potential sanitary risks. These findings confirm that the degradation of urban mangroves not only disrupts critical ecological functions but also facilitates the proliferation and dissemination of opportunistic fungi, some exhibiting resistance to antifungal agents, with direct implications for public health.

Physicochemical parameters such as slightly acidic pH, moderate eutrophication, and the presence of heavy metals suggest that microbial community reconfiguration occurs even under sub-extreme environmental conditions, driven by the synergistic effects of multiple stressors. The concurrent presence of symbiotic mycorrhizal fungi reflects that, despite degradation, some functional components of the ecosystem persist, highlighting the critical importance of mangrove conservation and restoration efforts.

Collectively, these data reinforce mangroves’ role as sentinel ecosystems, sensitive to environmental impacts and crucial for microbiological monitoring. Consequently, environmental management strategies should incorporate metagenomic surveillance to detect early shifts in fungal community composition indicative of ecological imbalance or epidemiological threat.

This study advances the understanding of microbial ecology in coastal environments under urban pressure and emphasizes the urgent need to integrate comprehensive taxonomic and functional fungal community analyses into public policies aimed at mangrove protection and sustainable management. Moreover, pilot studies of this nature are essential for anticipating the emergence of new fungal pathogens and resistance mechanisms, enabling early detection and the implementation of targeted mitigation strategies. By incorporating metagenomic surveillance into routine environmental monitoring, it becomes possible to proactively address potential ecological and public health risks, ensuring the long-term resilience and functionality of these critical ecosystems.

## Figures and Tables

**Table 1:** Summary of fungal community richness and sequencing depth obtained from sediment samples collected in a single impacted mangrove.

Sample	ASVs	Sequences (Reads)
Sample 1	1013	68,281
Sample 2	756	67,345
Total	1769	135,626

## Data Availability

Data are contained within the article.

## References

[1] Britto Martins De Oliveira J., Corrêa Junior D., Parente C.E.T., Frases S. (2025). Fungi in Mangrove: Ecological Importance, Climate Change Impacts, and the Role in Environmental Remediation. Microorganisms.

[2] Alongi D.M. (2002). Present state and future of the world’s mangrove forests. Environ. Conserv..

[3] Valiela I., Bowen J.L., York J.K. (2001). Mangrove Forests: One of the World’s Threatened Major Tropical Environments. BioScience.

[4] Moitinho M.A., Chiaramonte J.B., Bononi L., Gumiere T., Melo I.S., Taketani R.G. (2022). Fungal succession on the decomposition of three plant species from a Brazilian mangrove. Sci. Rep..

[5] Hyde K.D., Lee S.Y. (1995). Ecology of mangrove fungi and their role in nutrient cycling: What gaps occur in our knowledge?. Hydrobiologia.

[6] Marcial Gomes N.C., Borges L.R., Paranhos R., Pinto F.N., MendonÃ§a-Hagler L.C.S., Smalla K. (2008). Exploring the diversity of bacterial communities in sediments of urban mangrove forests: Sediment bacterial communities in urban mangrove forests. FEMS Microbiol. Ecol..

[7] Silva F.S.R., Da Silva Y.J.A.B., Maia A.J., Biondi C.M., Araújo P.R.M., Barbosa R.S., Silva C.M.C.A.C., Luiz T.C.S., Araújo A.F.V. (2023). Prediction of heavy metals in polluted mangrove soils in Brazil with the highest reported levels of mercury using near-infrared spectroscopy. Environ. Geochem. Health.

[8] Yang C., Zhao Y., Cao W., Xing M., Xu X., Wang Z., Sun J. (2022). Metagenomic analysis reveals antibiotic resistance genes and virulence factors in the saline-alkali soils from the Yellow River Delta, China. Environ. Res..

[9] Hau P.-T., Shiu A., Tam E.W.-T., Chau E.C.-T., Murillo M., Humer E., Po W.-W., Yu R.C.-W., Fung J., Seto S.-W. (2024). Diversity and Antifungal Susceptibilities of Yeasts from Mangroves in Hong Kong, China—A One Health Aspect. JoF.

[10] Xiao Y., He M., Xie J., Liu L., Zhang X. (2021). Effects of heavy metals and organic matter fractions on the fungal communities in mangrove sediments from Techeng Isle, South China. Ecotoxicol. Environ. Saf..

[11] Yi J., Lo L.S.H., Liu H., Qian P.-Y., Cheng J. (2021). Study of Heavy Metals and Microbial Communities in Contaminated Sediments Along an Urban Estuary. Front. Mar. Sci..

[12] Meng S., Peng T., Pratush A., Huang T., Hu Z. (2021). Interactions between heavy metals and bacteria in mangroves. Mar. Pollut. Bull..

[13] Shuaib M., Azam N., Bahadur S., Romman M., Yu Q., Xuexiu C. (2021). Variation and succession of microbial communities under the conditions of persistent heavy metal and their survival mechanism. Microb. Pathog..

[14] Fortunato J.M., Hypolito R., Moura C.L., Nascimento S.C. (2012). Caracterização da contaminação por metais pesados em área de mangüezal, Município de Santos (SP). Rev. Inst. Geol..

[15] Fontana L.F., Belart P., Bonetti C., Junior D.S., Frontalini F., Martínez-Colón M., Bouchet V.M.P., Laut L. (2024). Foraminifera and geomicrobiology as indicators of the environmental recovery in a mangrove affected by oil spills in the Guanabara Bay (Brazil). Sci. Total Environ..

[16] Rovai A.S., Twilley R.R., Worthington T.A., Riul P. (2022). Brazilian Mangroves: Blue Carbon Hotspots of National and Global Relevance to Natural Climate Solutions. Front. For. Glob. Change.

[17] Farias C.O., Hamacher C., Wagener A.D.L.R., Campos R.C.D., Godoy J.M. (2007). Trace metal contamination in mangrove sediments, Guanabara Bay, Rio de Janeiro, Brazil. J. Braz. Chem. Soc..

[18] Blankespoor B., Dasgupta S., Lange G.-M. (2017). Mangroves as a protection from storm surges in a changing climate. Ambio.

[19] Krishna S., Lemmen C., Örey S., Rehren J., Pane J.D., Mathis M., Püts M., Hokamp S., Pradhan H.K., Hasenbein M. (2025). Interactive effects of multiple stressors in coastal ecosystems. Front. Mar. Sci..

[20] Shi X., Zhou S., Xu L., Nethmini R.T., Zhang Y., Huang L., Dong K., Zhao H., Pan L. (2025). Shifts in Soil Fungal Community and Trophic Modes During Mangrove Ecosystem Restoration. JoF.

[21] MacPherson S., Akache B., Weber S., De Deken X., Raymond M., Turcotte B. (2005). *Candida albicans* Zinc Cluster Protein Upc2p Confers Resistance to Antifungal Drugs and Is an Activator of Ergosterol Biosynthetic Genes. Antimicrob. Agents Chemother..

[22] Seidel D., Wurster S., Jenks J.D., Sati H., Gangneux J.-P., Egger M., Alastruey-Izquierdo A., Ford N.P., Chowdhary A., Sprute R. (2024). Impact of climate change and natural disasters on fungal infections. Lancet Microbe.

[23] Merhaby D., Rabodonirina S., Net S., Ouddane B., Halwani J. (2019). Overview of sediments pollution by PAHs and PCBs in mediterranean basin: Transport, fate, occurrence, and distribution. Mar. Pollut. Bull..

[24] Gajewska J., Floryszak-Wieczorek J., Sobieszczuk-Nowicka E., Mattoo A., Arasimowicz-Jelonek M. (2022). Fungal and oomycete pathogens and heavy metals: An inglorious couple in the environment. IMA Fungus.

[25] Cabral L., Júnior G.V.L., Pereira de Sousa S.T., Dias A.C.F., Lira Cadete L., Andreote F.D., Hess M., de Oliveira V.M. (2016). Anthropogenic impact on mangrove sediments triggers differential responses in the heavy metals and antibiotic resistomes of microbial communities. Environ. Pollut..

[26] Metcalf R., Akinbobola A., Woodford L., Quilliam R.S. (2025). Thermotolerance, virulence, and drug resistance of human pathogenic Candida species colonising plastic pollution in aquatic ecosystems. Environ. Sci. Pollut. Res..

[27] Erazo N.G., Bowman J.S. (2021). Sensitivity of the mangrove-estuarine microbial community to aquaculture effluent. iScience.

[28] Zhao S., Sun Y., Su L., Yan L., Lin X., Long Y., Zhang A., Zhao Q. (2025). Significant Enrichment of Potential Pathogenic Fungi in Soil Mediated by Flavonoids, Phenolic Acids, and Organic Acids. JoF.

[29] Barroso G.C., Abril G., Machado W., Abuchacra R.C., Peixoto R.B., Bernardes M., Marques G.S., Sanders C.J., Oliveira G.B., Oliveira Filho S.R. (2022). Linking eutrophication to carbon dioxide and methane emissions from exposed mangrove soils along an urban gradient. Sci. Total Environ..

[30] Hou D., Huang Z., Zeng S., Liu J., Wei D., Deng X., Weng S., He Z., He J. (2017). Environmental Factors Shape Water Microbial Community Structure and Function in Shrimp Cultural Enclosure Ecosystems. Front. Microbiol..

[31] Laux M., Ciapina L.P., De Carvalho F.M., Gerber A.L., Guimarães A.P.C., Apolinário M., Paes J.E.S., Jonck C.R., De Vasconcelos A.T.R. (2024). Living in mangroves: A syntrophic scenario unveiling a resourceful microbiome. BMC Microbiol..

[32] Tavares T.C.L., Bezerra W.M., Normando L.R.O., Rosado A.S., Melo V.M.M. (2021). Brazilian Semi-Arid Mangroves-Associated Microbiome as Pools of Richness and Complexity in a Changing World. Front. Microbiol..

[33] Ghizelini A.M., Mendonça-Hagler L.C.S., Macrae A. (2012). Microbial diversity in Brazilian mangrove sediments: A mini review. Braz. J. Microbiol..

[34] Fistarol G.O., Coutinho F.H., Moreira A.P.B., Venas T., Cánovas A., de Paula S.E.M., Coutinho R., de Moura R.L., Valentin J.L., Tenenbaum D.R. (2015). Environmental and Sanitary Conditions of Guanabara Bay, Rio de Janeiro. Front. Microbiol..

[35] Vilela C.G., Figueira B.O., Macedo M.C., Baptista Neto J.A. (2014). Late Holocene evolution and increasing pollution in Guanabara Bay, Rio de Janeiro, SE Brazil. Mar. Pollut. Bull..

[36] De Carvalho Aguiar V.M., De Lima M.N., Abuchacra R.C., Abuchacra P.F.F., Neto J.A.B., Borges H.V., De Oliveira V.C. (2016). Ecological risks of trace metals in Guanabara Bay, Rio de Janeiro, Brazil: An index analysis approach. Ecotoxicol. Environ. Saf..

[37] Abreu F.E.L., Batista R.M., Zanardi-Lamardo E., Yogui G.T., Amado L.L., Ribeiro-Brasil D.R.G., Franco T.C.R.D.S., Viana J.L.M., Fernandez M.A., Castro I.B. (2025). Antifouling paint residues in areas impacted by maritime activities along 6000 km of Brazilian coastline. Sci. Total Environ..

[38] Do Carmo Fernández T., Souto-Neto J.A., Villar Freret-Meurer N., De Assis Machado L., Do Carmo Vaccani A., Dos Santos Cabiró G., Bezerra J.J.V., De Almeida R.F., Saint’Pierre T.D., Hauser-Davis R.A. (2025). First report on Technology-Critical Elements in seahorses from Rio de Janeiro, Southeastern Brazil. Mar. Pollut. Bull..

[39] World Reference Base|FAO SOILS PORTAL|Food and Agriculture Organization of the United Nations. https://www.fao.org/soils-portal/data-hub/soil-classification/world-reference-base/en/.

[40] Callahan B.J., McMurdie P.J., Rosen M.J., Han A.W., Johnson A.J.A., Holmes S.P. (2016). DADA2: High-resolution sample inference from Illumina amplicon data. Nat. Methods.

[41] Yao Y., Zhao J., Miao L., Hou J. (2022). Effects of sediment physicochemical factors and heavy metals on the diversity, structure, and functions of bacterial and fungal communities from a eutrophic river. Environ. Pollut..

[42] Jacob J.K.S., Witzel K., Dela Cruz T.E.E. (2023). Comparative Diversity and Functional Traits of Fungal Endophytes in Response to Elevated Mineral Content in a Mangrove Ecosystem. JoF.

[43] Ghizelini A.M., Martins K.G., Gießelmann U.C., Santoro E., Pasqualette L., Mendonça-Hagler L.C.S., Rosado A.S., Macrae A. (2019). Fungal communities in oil contaminated mangrove sediments—Who is in the mud?. Mar. Pollut. Bull..

[44] Bahram M., Hildebrand F., Forslund S.K., Anderson J.L., Soudzilovskaia N.A., Bodegom P.M., Bengtsson-Palme J., Anslan S., Coelho L.P., Harend H. (2018). Structure and function of the global topsoil microbiome. Nature.

[45] Tedersoo L., Bahram M., Põlme S., Kõljalg U., Yorou N.S., Wijesundera R., Villarreal Ruiz L., Vasco-Palacios A.M., Thu P.Q., Suija A. (2014). Fungal biogeography. Global diversity and geography of soil fungi. Science.

[46] Devadatha B., Jones E.B.G., Pang K.L., Abdel-Wahab M.A., Hyde K.D., Sakayaroj J., Bahkali A.H., Calabon M.S., Sarma V.V., Sutreong S. (2021). Occurrence and geographical distribution of mangrove fungi. Fungal Divers..

[47] Tedersoo L., Tooming-Klunderud A., Anslan S. (2018). PacBio metabarcoding of Fungi and other eukaryotes: Errors, biases and perspectives. New Phytol..

[48] Taghavi Ghasemkheili F., Pirdashti H., Ghanbary M.A.T., Emadi M., Ghadirnezhad Shiade S.R. (2021). Quantifying the Fungal Population Dynamics in Contaminated Soil Using Spatial Analysis. SSRN Electron. J..

[49] Rodriguez-Sanchez A., Tomasek A., McMillan S., Yufra S., Yupanqui M., Rondon R., Hoagland L. (2022). Composition and potential functional roles of soil fungal communities on arid farms in Arequipa (Southern Peru) characterized using SMRT sequencing. Appl. Soil. Ecol..

[50] de S Araújo G.R., de Souza W., Frases S. (2017). The Hidden Pathogenic Potential of Environmental Fungi. Future Microbiol..

[51] El-Sharkawy M., Alotaibi M.O., Li J., Du D., Mahmoud E. (2025). Heavy Metal Pollution in Coastal Environments: Ecological Implications and Management Strategies: A Review. Sustainability.

[52] Cabral L., Siqueira J.O., Soares C.R.F.S., Pinto J.E.B.P. (2010). Retenção de metais pesados em micélio de fungos micorrízicos arbusculares. Quím. Nova.

[53] Godoy M.D.P., Lacerda L.D.D. (2015). Mangroves Response to Climate Change: A Review of Recent Findings on Mangrove Extension and Distribution. An. Acad. Bras. Ciênc..

[54] Sridhar K.R. (2005). Diversity of fungi in mangrove ecosystems. Microbial Diversity: Current Perspectives and Potential Applications.

[55] Chynel M., Rockomanovic S., Abril G., Barroso G., Marotta H., Machado W., Sanders C.J., Thiney N., Meziane T. (2022). Contrasting organic matter composition in pristine and eutrophicated mangroves revealed by fatty acids and stable isotopes (Rio de Janeiro, Brazil). Estuar. Coast. Shelf Sci..

[56] Latgé J.-P., Chamilos G. (2019). Aspergillus fumigatus and Aspergillosis in 2019. Clin. Microbiol. Rev..

[57] Paulussen C., Hallsworth J.E., Álvarez-Pérez S., Nierman W.C., Hamill P.G., Blain D., Rediers H., Lievens B. (2017). Ecology of aspergillosis: Insights into the pathogenic potency of *Aspergillus fumigatus* and some other *Aspergillus* species. Microb. Biotechnol..

[58] Pappas P.G., Kauffman C.A., Andes D.R., Clancy C.J., Marr K.A., Ostrosky-Zeichner L., Reboli A.C., Schuster M.G., Vazquez J.A., Walsh T.J. (2016). Clinical Practice Guideline for the Management of Candidiasis: 2016 Update by the Infectious Diseases Society of America. Clin. Infect. Dis..

[59] Short B., Brown J., Delaney C., Sherry L., Williams C., Ramage G., Kean R. (2019). Candida auris exhibits resilient biofilm characteristics in vitro: Implications for environmental persistence. J. Hosp. Infect..

[60] Ramage G., Rajendran R., Sherry L., Williams C. (2012). Fungal Biofilm Resistance. Int. J. Microbiol..

[61] Perfect J.R. (2017). The antifungal pipeline: A reality check. Nat. Rev. Drug Discov..

[62] Baptista Neto J.A., Smith B.J., McAllister J.J. (2000). Heavy metal concentrations in surface sediments in a nearshore environment, Jurujuba Sound, Southeast Brazil. Environ. Pollut..

[63] Zhang Z., Zhou J., Song J., Wang Q., Liu H., Tang X. (2017). Habitat suitability index model of the sea cucumber Apostichopus japonicus (Selenka): A case study of Shandong Peninsula, China. Mar. Pollut. Bull..

[64] Kim K.-J., Lee J., Wang W., Lee Y., Oh E., Park K.-H., Park C., Woo G.-E., Son Y.-J., Kang H. (2021). Austalide K from the Fungus Penicillium rudallense Prevents LPS-Induced Bone Loss in Mice by Inhibiting Osteoclast Differentiation and Promoting Osteoblast Differentiation. Int. J. Mol. Sci..

